# Supplementary figures and images for: A geographically weighted random forest approach for evaluate forest change drivers in the Northern Ecuadorian Amazon
Source: PLoS One. 2019 Dec 23;14(12):e0226224. doi: 10.1371/journal.pone.0226224 (PMC6927660; doi:10.1371/journal.pone.0226224)

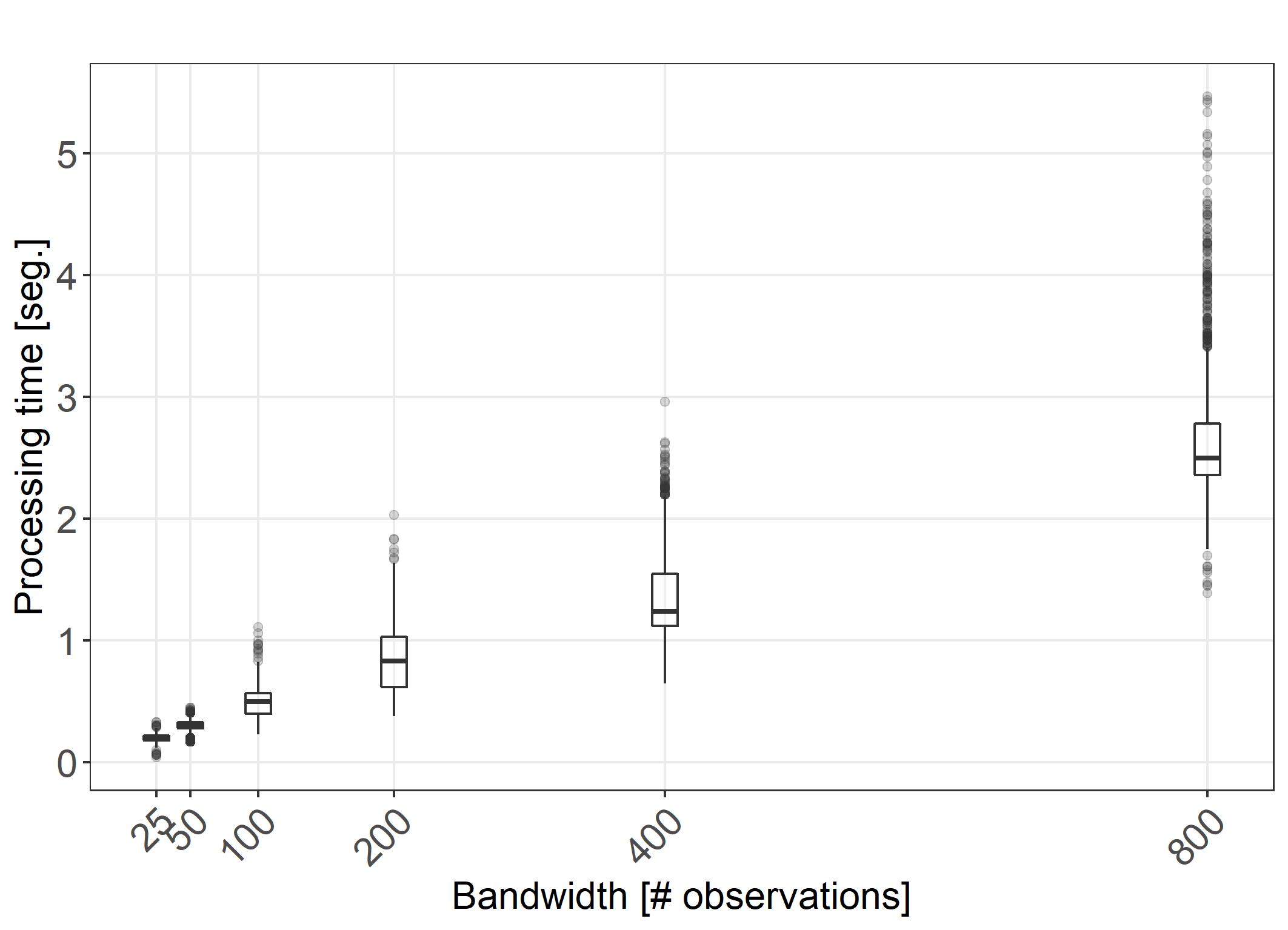

Supplement: S1 Appendix — (JPG) [file pone.0226224.s001.jpg]

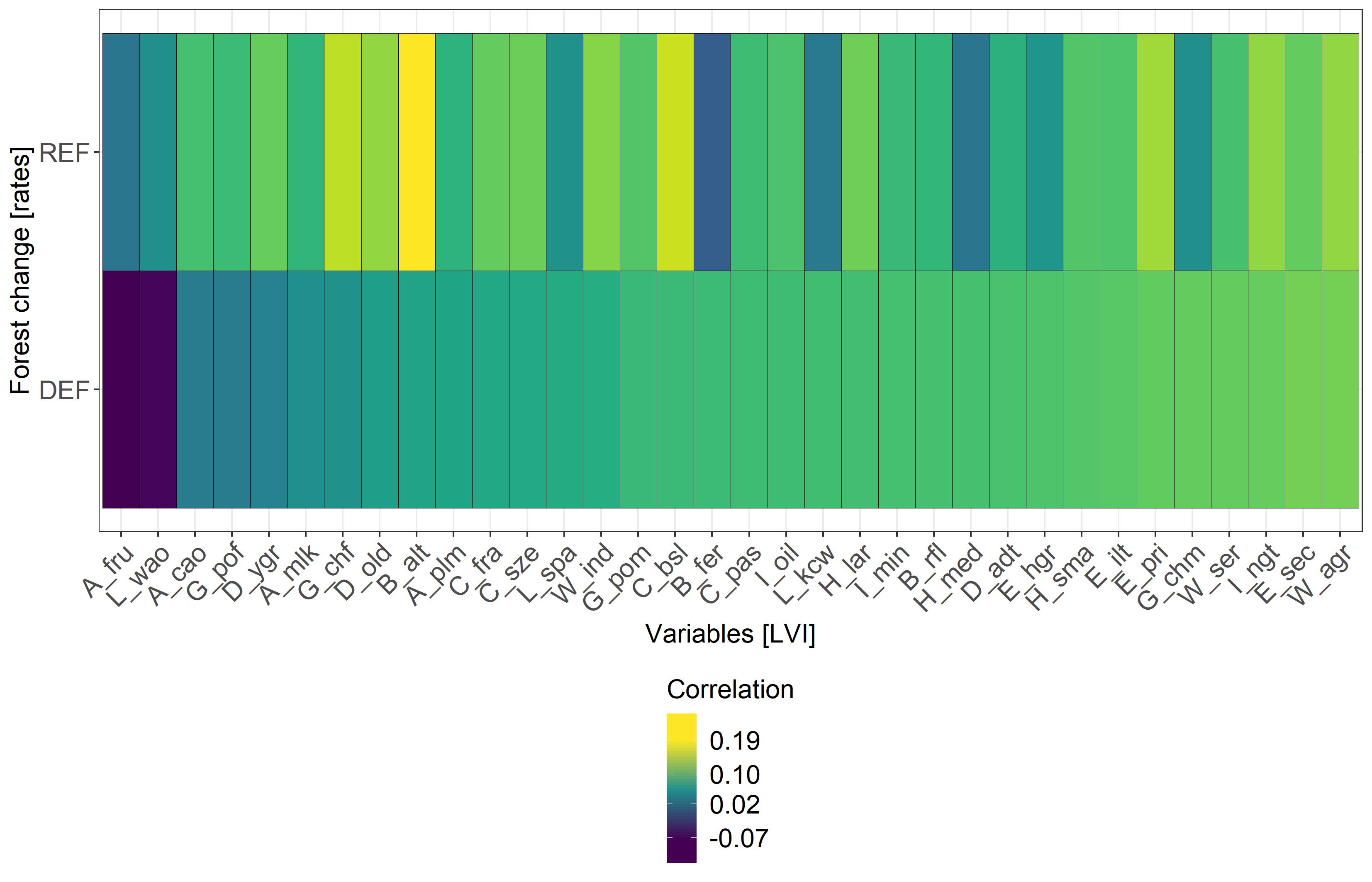

Supplement: S2 Appendix — (JPG) [file pone.0226224.s002.jpg]
