# Supplementary material for: A geographically weighted random forest approach for evaluate forest change drivers in the Northern Ecuadorian Amazon
Source: PLoS One. 2019 Dec 23;14(12):e0226224. doi: 10.1371/journal.pone.0226224 (PMC6927660; doi:10.1371/journal.pone.0226224)
Supplement: S3 Appendix — https://github.com/FSantosCodes/GWRFC. (DOCX) [file pone.0226224.s003.docx]

Appendix 3. Source code of GWRFC algorithm

<https://github.com/FSantosCodes/GWRFC>
